# Image quality of abdominal photon-counting CT with reduced contrast media dose: Evaluation of reduced contrast media protocols during the COVID19 pandemic supply shortage

**DOI:** 10.1016/j.heliyon.2024.e28142

**Published:** 2024-03-18

**Authors:** Yannik C. Layer, Alexander Isaak, Narine Mesropyan, Patrick A. Kupczyk, Julian A. Luetkens, Tatjana Dell, Ulrike I. Attenberger, Daniel Kuetting

**Affiliations:** Department of Diagnostic and Interventional Radiology, University Hospital Bonn, Venusberg-Campus 1, 53127, Bonn, Germany

**Keywords:** Contrast media, Photon-counting detector computed tomography, Dose reduction, Iodine signal, Abdominal imaging

## Abstract

**Rationale and objectives:**

Aim of this study was to assess the impact of contrast media dose (CMD) reduction on diagnostic quality of photon-counting detector CT (PCD-CT) and energy-integrating detector CT (EID-CT).

**Methods:**

CT scans of the abdominal region with differing CMD acquired in portal venous phase on a PCD-CT were included and compared to EID-CT scans. Diagnostic quality and contrast intensity were rated. Additionally, readers had to assign the scans to reduced or regular CMD. Regions-of-interest (ROIs) were placed in defined segments of portal vein, inferior vena cava, liver, spleen, kidneys, abdominal aorta and muscular tissue. Signal-to-noise ratio (SNR) and contrast-to-noise ratio (CNR) were calculated.

**Results:**

Overall 158 CT scans performed on a PCD-CT and 68 examinations on an EID-CT were analyzed. Overall diagnostic quality showed no significant differences for PCD-CT with standard CMD which scored a median 5 (IQR:5-5) and PCD-CT with 70% CMD scoring 5 (4–5). (For PCD-CT, 71.69% of the examinations with reduced CMD were assigned to regular CMD by the readers, for EID-CT 9.09%. Averaged for all measurements SNR for 50% CMD was reduced by 19% in PCD-CT (EID-CT 34%) and CNR by 48% (EID-CT 56%). Virtual monoenergetic images (VMI)_50keV_ for PCD-CT images acquired with 50% CMD showed an increase in SNR by 72% and CNR by 153%.

**Conclusions:**

Diagnostic interpretability of PCD-CT examinations with reduction of up to 50% CMD is maintained. PCD-CT deducted scans especially with 70% CMD were often not recognized as CMD reduced scans. Compared to EID-CT less decline in SNR and CNR is observed for CMD reduced PCD-CT images. Employing VMI_50keV_ for CMD-reduced PCD-CT images compensated for the effects.

## 1 Introduction

In 2005, the annual usage of iodinated intravascular contrast media was applied in over 75 million procedures [1]. The prevalence of computed tomography (CT) examinations has risen significantly in recent decades, with the United States alone conducting 84 million CT scans in 2016, indicating a corresponding increase in the use of intravenous iodinated contrast media [2].

Reducing the administration of contrast agents has been a persistent focus over the past few decades, driven by medical considerations such as the risk of contrast-induced nephropathy, hyperthyroidism, and anaphylactic reactions, as well as environmental and financial factors [[3], [4], [5]]. Moreover, the COVID-19 pandemic has further prompted the reduction of contrast agent usage, as limited availability necessitated dose reduction in many healthcare institutions [6,7]. Consequently, our institution implemented a reduced contrast media dose (CMD) protocol to conserve contrast agent volume while ensuring diagnostic efficacy.

An established concept for the reduction of contrast media is low kV image acquisition which takes advantages of the increase of iodine attenuation when approaching the iodine k-edge of 33 keV [8]. The same concept is used in dual-energy derived low-energy virtual monoenergetic images [9]. In contrast to dual-energy energy-integrating detector CT, photon-counting detector CT uses a direct conversion of photons into electrical signals combined with more sensitive and accurate photon detection which simultaneously allows spectral evaluation [9].

This study aimed to evaluate the diagnostic efficacy of photon-counting detector CT (PCD-CT) images acquired with reduced CMD.

## 2 Materials and methods

The study was approved by the local ethics review board (Ethics Committee of the Medical Faculty of the University of Bonn; listed under file reference 027/23) and the need of informed consent was waived. As every scan was clinically acquired, no scan was performed exclusively for research purpose. The study is in compliance with the Declaration of Helsinki and its amendments.

In a single-center, retrospective study design, patients receiving an abdominal CT scan in a portal venous phase between August 2022 and September 2022 were included in this study. Patients were examined for various clinical indications on a photon-counting detector CT (NAEOTOM Alpha, Siemens Healthcare GmbH, Erlangen, Germany) and an energy-integrating detector CT (iCT 256, Philips Healthcare, Best, The Netherlands).

### 2.1 Imaging protocol

Patients were placed in head-first-supine position. For PCD-CT, the following scan parameters were used: tube voltage of 120 kVp with activated automatic tube current modulation; pitch of 0.8; 0.5 s gantry rotation time. Collimation was 144 x 0.4 mm. Slice thickness of the reconstruction was set to 1 mm and an increment of 0.7 mm was employed. For image reconstruction a standard body kernel (Br40; Siemens Healthcare GmbH, Erlangen, Germany) and Quantum Iterative Reconstruction (QIR Level 3; Siemens Healthcare GmbH, Erlangen, Germany) were used. For energy-integrating detector CT (EID-CT), scan parameters were a tube voltage of 120 kVp with activated automatic tube current modulation, a pitch of 0.93 and a gantry rotation time of 0.4 s. Collimation was 128 x 0.625 mm. Reconstruction parameters were 2 mm slice thickness of the reconstruction with an increment of 1 mm. iDose (level 3, Philips Healthcare, Best, The Netherlands) was used for image reconstruction.

Intravenous administration of iodine-based contrast agent (Accupaque 300 mg/ml, GE Healthcare Buchler GmbH & Co. KG) was performed using a monophasic bolus technique. The employed flow rate was 3 ml/s. A bolus of 40 ml of physiologic saline solution was administered afterwards at the same flow rate. A weight-adaptation approach was employed, with the volume of contrast media ranging from 60 to 80 ml depending on the patients' body mass index (BMI). Patients with a BMI below 20 received 60 ml contrast media, patients with a BMI between 20 and 30 received 70 ml and patients with a BMI above 30 received 80 ml of the contrast media.

To address the shortage of contrast media, patients were categorized into different groups and received varying doses: 100% CMD, 70% CMD, or 50% CMD, based on the severity of the shortage. In the subsequent examination, a circular region of interest (ROI) was placed in the descending aorta. The scan was initiated when the attenuation within the ROI reached a threshold of 120 Hounsfield Units (HU), with a post-threshold delay of 60 s. Virtual monoenergetic images (VMI) were reconstructed using the vendor's regular software (Syngo.Via VB70, Siemens Healthcare GmbH, Erlangen, Germany).

### 2.2 Quantitative image analysis

For quantitative assessment ROIs were placed in the following abdominal regions: abdominal aorta, inferior vena cava, portal vein, left and right lobe of the liver, spleen, cortex of both kidneys, parenchyma of both kidneys and the left and right psoas muscle. ROI size was as large as possible, ensuring that lesions or larger vessels were not included. Placement of ROIs was conducted using a regular clinical DICOM viewer (Deep Unity R20 XX; Dedalus HealthCare GmbH, Bonn, Germany). Images were presented with a slice thickness of 1 mm. Values and standard deviation of attenuation were evaluated and averaged for matched measurements (e.g. right and left liver lobe). Based on this, signal-to-noise ratio (SNR= HU_target_/standard deviation_target_ (SD_target_)) and contrast-to-noise ratio (CNR= (HU_target_ – HU_muscle_/SD_muscle_) were calculated.

### 2.3 Qualitative image analysis

Qualitative assessment was evaluated by two radiologists with two (YCL) and five (AI) years of experience in CT imaging. Both raters assessed CT images regarding contrast intensity and overall image quality using a five-point Likert scale for each criteria. The rating was defined as follows: (1) non-diagnostic; (2) poor, insufficient diagnostic confidence; (3) moderate, low diagnostic confidence; (4) good, diagnostic with high confidence; and (5) excellent, full diagnostic confidence. Additionally, readers categorized examinations as either regular or reduced CMD. The readers were blinded to technical data and rated the acquired images in random order.

### 2.4 Statistical analysis

For Statistical analyses IBM SPSS Version 27 (IBM Corp., Armonk, NY, USA) was used. Graphs were plotted using GraphPad PRISM Version 6.02 (GraphPad Software, San Diego, CA, USA). Quantitatively, results are expressed as mean and standard deviation. Shapiro-Wilk test, Mann-Whitney test and unpaired *t*-test were used for statistical analysis of qualitative and quantitative image parameters. Qualitative results are stated as median with interquartile range (IQR). The intraclass correlation coefficient (ICC) was used for the assessment of interrater reliability. ICC estimates and their 95% confident intervals (CI) were calculated on the basis of a mean-rating (k = 2), consistency, two-way mixed-effects model. P-values below 0.01 were defined as significant.

## 3 Results

### 3.1 Participant characteristics

**Fig. 1:**
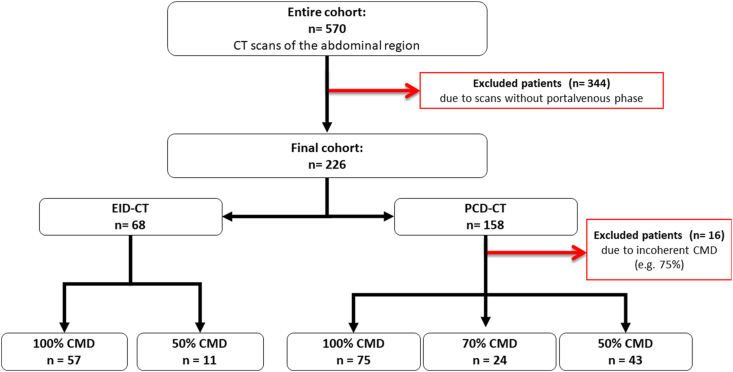
The patient flow chart shows exclusion criteria and the assignment of patients scanned in portal venous contrast media phase on photon-counting detector CT (PCD-CT) or energy-integrating detector CT (EID-CT) to differing contrast media doses (CMD).

Between August 2022 and September 2022, 570 patients received scans of the abdominal region on one the two institutional CT scanners. Of these examinations 226 were performed in portal venous phase (PCD-CT: 158; EID-CT: 68). 83 patients of the PCD-CT cohort received a reduced CMD whereas 75 patients received standard CMD. Of the 83 patients scanned with reduced CMD at PCD-CT, 24 received 70% CMD and 43 50% CMD, the other 16 patients were excluded as they received differing fractions of the CMD, e.g. 75% or 60%. 11 patients received a portal venous abdominal scan with reduced CMD on an EID-CT and 57 patients received a regular CMD scan. Fig. 1 shows the patient flow chart. Overall, 139 male and 87 female patients were included in the analysis, resulting in a median age of 64 (56–73) years. Median age was 64.5 (56–73.75) years for PCD-CT and 63 (59–72) years for EID-CT, respectively. Mean BMI was 27.18 kg/m^2^ for PCD-CT and 24.73 kg/m^2^ for EID-CT (p < 0.05). Mean dose length product (DLP) on PCD-CT was 417.54 mGy*cm (EID-CT: 548.51 mGy*cm; p < 0.05) and mean CTDI_vol_ was 6.57 mGy (EID-CT: 8.64 mGy; p < 0.05). Mean contrast media volume applied on PCD-CT was 71.41 ml (EID-CT 76.93 ml) for regular CMD and 35.45 ml for 50% CMD (EID-CT 35.91 ml). Table 1 gives an overview of the participant characteristics.

**Table 1:** Summary of the patients‘ characterstics interest for photon-counting detector CT (PCD-CT) and energy-integrating detector CT (EID-CT).

	PCD-CT (n = 158)	EID-CT (n = 68)	Overall (n = 226)
Age (median (range); years)	64.5 (24–91)	63 (20–89)	64 (20–91)
Male (n = )	99	40	139
BMI (kg/m^2^)	27.18 ± 6.41	24.73 ± 5.53	26.37 ± 6.24
DLP (mGy*cm)	417.54 ± 191.87	548.51 ± 270.98	460 ± 228.59
CTDI_vol_ (mGy)	6.57 ± 2.59	8.64 ± 4.58	7.24 ± 3.50

### 3.2 Quantitative image analysis

**Fig. 2:**
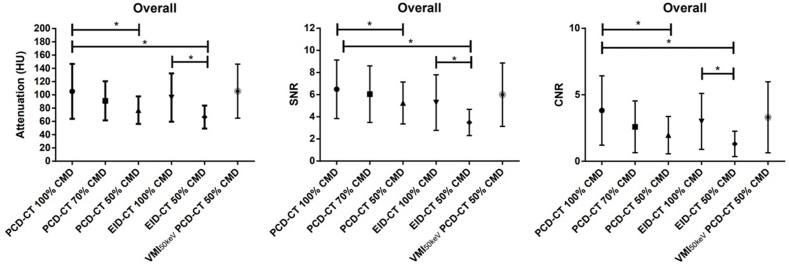
Mean overall attenuation (left panel), signal-to-noise ratio (SNR) (center panel), and contrast-to-noise ratio (CNR) (right panel) and corresponding standard deviation within defined regions of interest plotted for photon-counting detector CT (PCD-CT) at 100% contrast media dose (CMD), PCD-CT at 70% CMD, PCD-CT at 50% CMD, energy-integrating detector CT (EID-CT) at 100% CMD, EID-CT at 50% CMD and virtual monoenergetic images (VMI) at 50 keV of PCD-CT with 50% CMD. P-values below 0.01 are indicated with an asterisk.

Results of quantitative image analysis are summarized in Table 2, Table 3, Table 4. Attenuation, SNR and CNR decreased for all investigated organs with reduction of contrast media volume, independent of the employed CT scanner. No differences were observed between attenuation measurements in the psoas muscle. In comparison to EID-CT, PCD-CT showed higher attenuation, SNR and CNR at regular CMD (Fig. 2, Table 3, Table 4) and reduced CMD (Table 3, Table 4). In detail, with PCD-CT SNR of the liver at 50% CMD was reduced by 13.89% and CNR by 35.74% compared to standard CMD. In PCD-CT SNR of the liver at 70% CMD was reduced by 2.81% and CNR by 28.16% compared to standard CMD. SNR of EID-CT acquired with 50% CMD declined by 24.45% and CNR by 49.75% compared to regular contrast media volumes. Averaged over all measurements, SNR of PCD-CT at 70% CMD was reduced by 6.84% (CNR: 32.13%) at 50% CMD by 19.06% (CNR: 48.44%). SNR of EID-CT acquired with 50% CMD was reduced by 34% (CNR: 56.44%). There were no statistically significant differences between SNR of PCD-CT with 100% CMD and 70% CMD (overall p = 0.0582). Employing VMI _50 keV_ for PCD-CT images acquired with 50% CMD lead to an increase in SNR by 72% and CNR by 153% compared to polychromatic PCD-CT images acquired with 50% CMD.

**Table 2:** Mean attenuation values and standard deviation within defined regions of interest for photon-counting detector CT (PCD-CT) and energy-integrating detector CT (EID-CT) at different contrast media dosage (CMD). Bold results were considered significant compared to PCD-CT with 100% CMD (p < 0.01).

	n	Aorta	Inferior vena cava	Portal vein	Liver	Spleen	Renal parenchyma	Renal cortex	Muscle
PCD-CT 100% CMD	75	130.78 ± 16.42	84.58 ± 17.42	151.26 ± 18.14	95.76 ± 15.63	102.34 ± 16.17	77.84 ± 17.20	145.48 ± 17.26	55.25 ± 16.06
PCD-CT 70% CMD	24	**107.54** ± 15.93	77.13 ± 15.83	**129.41** ± 16.15	**86.48** ± 14.58	**88.61** ± 15.33	69.72 ± 17.02	**112.73** ± 15.49	55.49 ± 16.65
PCD-CT 50% CMD	43	**88.37** ± 15.48	**71.27** ± 15.64	**99.04** ± 15.56	**75.61** ± 14.41	**77.1 ± 14.79**	**60.64** ± 14.59	**89.78** ± 15.52	51.88 ± 15.47
PCD-CT 50% CMD VMI 50 keV	43	131.39 ± 19.17	95.51 ± 19.50	154.59 ± 19.18	91.94 ± 16.97	100.16 ± 18.11	87.38 ± 18.22	133.38 ± 20.72	58.56 ± 17.83
EID-CT 100% CMD	57	122.94 ± 20.45	85.5 ± 20.85	**128.82 ± 20.52**	87.46 ± 17.24	**86.10 ± 17.33**	83.51 ± 19.23	**119.78 ± 19.32**	**48.61 ± 19.83**
EID-CT 50% CMD	11	**76.44** ± 21.35	**64.05** ± 21.5	**71.99** ± 22.9	**66.06** ± 17.76	**66.93** ± 18.43	69.79 ± 20.84	**71.45** ± 20.69	**45.19 ± 20.81**

**Table 3:** Mean signal-to-noise ratio (SNR) and standard deviation (SD) within defined regions of interest for photon-counting detector CT (PCD-CT) and energy-integrating detector CT (EID-CT) at different contrast media dosage (CMD). SNR was calculated as target attenuation values/target SD. Bold results were considered significant compared to PCD-CT with 100% CMD (p < 0.01).

	n	Aorta	Inferior vena cava	Portal vein	Liver	Spleen	Renal parenchyma	Renal cortex	Muscle
PCD-CT 100% CMD	75	8.30 ± 2.44	5.05 ± 1.51	8.68 ± 2.77	6.36 ± 1.64	6.63 ± 1.66	4.75 ± 1.41	8.68 ± 2.30	3.54 ± 0.77
PCD-CT 70% CMD	24	7.03 ± 2.11	5.17 ± 2.14	8.35 ± 2.78	6.18 ± 1.79	6.09 ± 1.92	4.35 ± 1.49	7.70 ± 2.75	3.53 ± 1.04
PCD-CT 50% CMD	43	**5.97 ± 1.70**	4.77 ± 1.42	**6.67 ± 2.19**	5.47 ± 1.65	**5.33 ± 1.20**	4.25 ± 1.28	**6.10 ± 2.25**	3.52 ± 0.99
PCD-CT 50% CMD VMI 50 keV	43	7.37 ± 2.54	5.29 ± 2.01	8.57 ± 3.13	5.76 ± 2.05	5.76 ± 1.66	4.93 ± 1.91	6.70 ± 4.09	3.49 ± 1.28
EID-CT 100% CMD	57	**6.58 ± 2.61**	4.52 ± 2.09	**6.65 ± 2.68**	**5.39 ± 2.02**	**5.37 ± 1.83**	4.58 ± 1.86	**6.60 ± 2.70**	**2.62 ± 0.91**
EID-CT 50% CMD	11	**3.76 ± 0.84**	**3.20 ± 1.00**	**3.45 ± 1.36**	**4.08 ± 0.90**	**3.85 ± 0.97**	3.48 ± 1.68	**3.82 ± 1.10**	**2.30 ± 0.68**

**Table 4:** Mean contrast-to-noise ratio (CNR) and standard deviation (SD) within defined regions of interest for photon-counting detector CT (PCD-CT) and energy-integrating detector CT (EID-CT) at different contrast media dosage (CMD). CNR was calculated as (target attenuation - muscle attenuation)/muscle SD. Bold results were considered significant compared to PCD-CT with 100% CMD (p < 0.01).

	n	Aorta	Inferior vena cava	Portal vein	Liver	Spleen	Renal parenchyma	Renal cortex
PCD-CT 100% CMD	75	4.95 ± 2.03	2.00 ± 1.51	6.46 ± 2.71	2.74 ± 1.09	3.09 ± 1.23	1.55 ± 1.12	5.91 ± 2.71
PCD-CT 70% CMD	24	**3.28 ± 1.42**	1.48 ± 1.21	**4.67 ± 2.42**	**1.97 ± 0.98**	**2.10 ± 0.93**	0.99 ± 0.59	**3.64 ± 2.26**
PCD-CT 50% CMD	43	**2.46 ± 1.11**	1.31 ± 0.82	**3.17 ± 1.43**	**1.76 ± 0.82**	**1.67 ± 0.81**	**0.75 ± 0.97**	**2.64 ± 1.86**
PCD-CT 50% CMD VMI 50 keV	43	4.37 ± 2.14	2.15 ± 1.21	5.70 ± 3.05	2.00 ± 1.02	2.49 ± 1.50	1.75 ± 1.85	4.59 ± 3.80
EID-CT 100% CMD	57	**4.09 ± 2.05**	2.07 ± 1.46	**4.34 ± 2.58**	2.31 ± 1.24	**2.32 ± 1.39**	1.95 ± 1.54	**3.89 ± 2.44**
EID-CT 50% CMD	11	**1.57 ± 0.61**	**0.94 ± 0.54**	**1.40 ± 0.78**	**1.16 ± 0.52**	**1.10 ± 0.57**	1.62 ± 1.93	**1.34 ± 0.93**

### 3.3 Qualitative image analysis

**Fig. 3:**
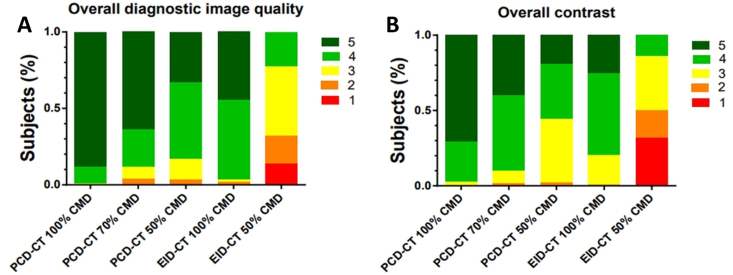
Bar plots show distribution of overall diagnostic quality rating (A) and overall contrast (B) rating for photon-counting detector CT (PCD-CT) at 100% contrast media dose (CMD), PCD-CT at 70% CMD, PCD-CT at 50% CMD, energy-integrating detector CT (EID-CT) at 100% CMD and EID-CT at 50% CMD.

Results of qualitative image analysis are summarized in Table 5 and Fig. 3A and B. Diagnostic quality of all PCD-CT scans performed with 70% CMD as well as 50% CMD was preserved in comparison to full contrast scans (PCD-CT 100% CMD 5 (5-5); 70% CMD 5 (4–5; p = 0.024); PCD-CT 50% CMD: 4 (4–5), p < 0.01; EID-CT 100% CMD: 4 (4–5), <0.01), whereas diagnostic quality of EID-CT at 50% CMD was markedly reduced (EID-CT 50% CMD 3 (2–3), p < 0.01).

**Table 5:** Median and inter-quartile range (IQR) of qualitative image ratings. Intraclass correlation coefficient (ICC) estimates and their 95% confidence intervals (CI) were calculated. ICC calculation is based on a mean-rating (k = 2), consistency, two-way mixed-effects model. Bold results were considered significant compared to photon-counting detector CT (PCD-CT) with 100% contrast media dose (CMD) (p < 0.01).

	PCD-CT 100% CMD	PCD-CT 70% CMD	PCD-CT 50% CMD	EID-CT 100% CMD	EID-CT 50% CMD	ICC
Contrast intensity	5 (4–5)	**4** (4–5)	**4** (3–4)	**4** (4–4.75)	**2.5** (1–3)	0.864 (95% CI: 0.823–0.895)
Overall diagnostic quality	5 (5–5)	5 (4–5)	**4** (4–5)	**4** (4–5)	**3** (2–3)	0.808 (95% CI: 0.750–0.852)
Classified 100% CMD	96%	90%	**58.51%**	86.84%	**9.09%**	0.857 (95% CI: 0.814–0.890)

Reduction of contrast intensity was less pronounced in PCD-CT (PCD-CT 100% CMD: 5 (4–5); PCD-CT 70% CMD: 4 (4–5); PCD-CT 50% CMD: 4 (3–4), p < 0.01) than in EID-CT (EID CT 100% CMD: 4 (4–4.75); EID-CT 50% CMD: 2.5 (1–3), p < 0.01).

**Fig. 4:**
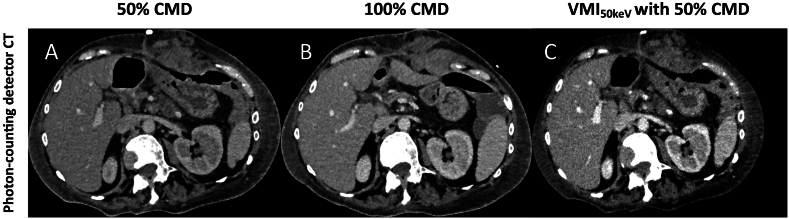
Representative axial images (window width/window level: 300/40 HU) of the same patient with 50% contrast media dose (CMD) (A), 100% CMD (B) and virtual monoenergetic images of 50% CMD at 50 keV (C) acquired with a photon-counting detector CT (PCD-CT).

**Fig. 5:**
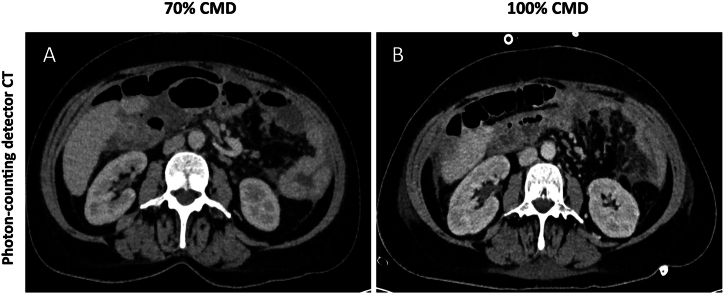
Representative axial images (window width/window level: 300/40 HU) of the same patient with 70% contrast media dose (CMD) (A) and 100% CMD (B) acquired with a photon-counting detector CT (PCD-CT).

90% of PCD-CT scans with 70% CMD and 58.51% of scans with 50% CMD were categorized as standard CMD by the raters (100% CMD PCD-CT: 96%), whereas only 9.09% of the EID-CT scans with 50% CMD were classified as standard CMD (EID-CT 100%: 86.84%). Representative images are shown in Fig. 4 A-C, 5 A/B and 6 A/B. The overall ICC was 0.844 (95% CI: 0.813–0.871), considered as good interrater agreement.ICC was 0.808 (95% CI: 0.750–0.852) for diagnostic quality, 0.864 (95% CI: 0.823–0.895) for contrast and 0.857 (95% CI: 0.814–0.890) for CMD classification, respectively (see Fig. 5).

## 4 Discussion

This is the first clinical investigation, as far as our knowledge extends, focusing on the evaluation of contrast media dose reduction in abdominal photon-counting detector CT. The primary outcome of this study reveals that although there is a decline in image quality with the reduction of contrast media volume, the diagnostic quality achieved with 70% CMD in PCD-CT is preserved. Furthermore, the image quality obtained in PCD-CT with 70% CMD surpasses that of EID CT with 100% CMD. Scans conducted with 70% CMD exhibited only marginal disparities in contrast-to-noise ratio (CNR) when compared to regular CMD, and they performed equally well in terms of signal-to-noise ratio (SNR) and overall image quality. Notably, the utilization of VMI at 50 keV effectively counteracted the effects of CMD reduction while preserving the integrity of high-quality imaging (Fig. 4).

Previous research indicates that the application of dual-energy CT with virtual low voltage image reconstruction offers a feasible means of reducing the usage of iodinated contrast media, without compromising image quality [8]. This is attributed to the fact that the iodine signal intensifies at lower kiloelectron volt (keV) levels, as the energy spectrum approaches the iodine K-edge of 33 keV [9]. Nonetheless, the utilization of this approach involves a tradeoff between increased image noise and radiation dose [10].

In 2021 the first PCD-CT was approved for clinical use [11]. This innovative technology provides inherent advantages over EID-CT, including decreased image noise, the potential for radiation dose reduction, improved spatial resolution, and the availability of artifact reduction options at high-energy levels [9].

**Fig. 6:**
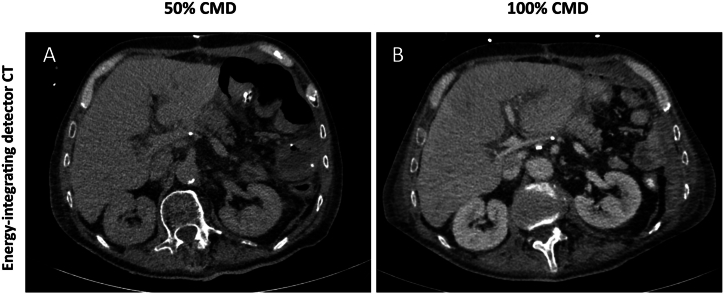
Representative axial images (window width/window level: 350/60 HU) of the same patient with 50% contrast media dose (CMD) (A) and 100% CMD (B) acquired with an energy-integrating detector CT (EID-CT). The images with 50% CMD were considered non-diagnostic.

Preliminary phantom studies have demonstrated the potential of PCD-CT in achieving CMD reductions of up to 37% without reducing signal intensities [12]. In PCD-CT images, the iodine signal is enhanced compared to EID-CT due to the direct registration of photons. In PCD-CT, both low-energy and high-energy photons contribute equally to the signal, leading to improved iodine signal representation. This capability of capturing and utilizing low-energy photons effectively contributes to the overall image quality and diagnostic accuracy of PCD-CT in evaluating iodinated contrast media [13]. The feature of enhanced iodine contrast extends the possibilities of low-kV imaging with increased iodine signal close to the iodine k-edge, a strategy widely used for reduction of contrast media volume nowadays; VMI reconstruction is a further option for iodine signal improvement in reduced contrast media protocols [14]. In our study, VMI at 50 keV proved to efficiently recover iodine signal without a reduction in SNR and CNR Therefore, low-energy VMI provide a legitimate option for the reduction of contrast media use (see Fig. 6).

The registration of every single photon combined with the use of energy thresholds and bins allows for energy discrimination and thus for the removal of background noise [13]. By applying an energy threshold value above the electrical noise level, the background noise can be filtered out from the signal. This explains why overall SNR and CNR were significantly higher for PCD-CT at 100% CMD and also at 70% CMD compared to EID-CT with 100% CMD. Increased SNR and CNR of PCD-CT had been reported in previous studies using regular CMD, but was not expected for 70% CMD [15]. Additionally we observed an overall radiation dose reduction of up to 35% for PCD-CT in comparison to EID-CT even though BMI was significantly higher for PCD-CT patients, which is in line with previous clinical studies for abdominal PCD-CT reporting around 32% reduction in an oncologic cohort and a 24% reduction in obese patients [15,16].

Our clinical results are in concordance with previous phantom studies, stating that CMD reduction of up to 37% is feasible in PCD-CT imaging [12]. In another phantom study Emrich et al. recently reported sufficient diagnostic image quality for a PCD-CT coronary angiography with a CMD reduction of up to 50% [17]. A recent PCD-CT clinical study demonstrated non-inferior image quality for thoracoabdominal CT angiography with 25% CMD reduction when using VMI [18]. The prospective study included 100 patients undergoing CTA with PCD-CT of the thoracoabdominal aorta; previous CTAs on an EID-CT served as a comparison. The results are consistent with those from the current study, stating a higher image-quality compared to EID-CT at equal CMD [18]. However, the study solely focussed on arterial vascular contrast; the parenchymal image quality of abdominal organs was not investigated. The current study could demonstrate image quality superiority of PCD-CT acquired with 70% CMD in comparison to EID-CT with full dose CMD with lower radiation doses in the PCD-CT collective. Moreover, for PCD-CT acquired with 50% CMD, SNR of the portal vein and liver, which are of particular relevance in oncologic reporting, did not differ from EID-CT with 100% CMD, further highlighting the higher iodine sensitivity of PCD-CT.

In this retrospective study, contrast media volumes were reduced due to a global supply shortage in iodinated contrast media. Although the dramatic scarcity has since been compensated, there is still a need to establish contrast media reduced protocols in preparation for possible future supply chain disruptions [19]. Apart from this, iodinated contrast agents are restrictively handled especially due to the risk of renal complications [20]. Even though the risk of contrast-induced acute kidney injury was overestimated in the past, contrast media should not be applied without caution; protocols should be adapted to body weight and the employed scanner [21,22]. Other complications of iodinated contrast media application include allergic like reactions, up to anaphylactic shock and iodine-induced hyperthyroidism [3]. Although severe reactions are rare, due to the frequent application severe adverse reactions are part of the clinical routine. Patients with impaired renal function could profit from reduced CMD. Cost savings are an additional incentive for reducing contrast media protocols [23]. Furthermore, there is a need for reduction of contrast agent volume in clinical protocols not only because of toxicity and risk of allergic reactions, but also due to environmental and sustainability aspects [5]. Iodinated contrast agents are increasingly found in drinking water sources and their breakdown products are deemed to be toxic [4,24]. As iodinated contrast media contamination of drinking water constitutes a major global environmental issue there is an urgent need for reduction of iodinated contrast agents, especially as currently there is no established method for their removal [25,26].

Limitations of this study are the retrospective and single-centered design. Further, ideally prospective, analysis of larger patient cohorts are necessary to optimally define weight adapted CMD protocols for PCD-CT. CT systems from 2 different vendors were compared, with different image reconstruction kernels and different image reconstruction techniques. This might lead to a different image impression which implicates potential reading bias. As a result of poor objective performance of EID-CT, clinical scanning with reduced CMD was stopped shortly after initiation, thus, the number of examinations with reduced CMD at EID-CT is limited. Indications for included scans varied, thus additional analysis of diagnostic markers (e.g. lesion detectability) was not possible. Furthermore, the cohorts were not matched for body weight, leading to a higher BMI in the PCD-CT cohort. Apart from contrast media volume, contrast signal in parenchymal tissues is also dependent on circulatory parameters such as cardiac output [27]. These factors were not considered. However, portal venous phase was visually verified before inclusion into the study.

## 5 Conclusions

Reducing iodine contrast media volume by 30% is possible in abdominal PCD-CT imaging without affecting image quality. A 50% reduction of contrast media volume leads to a reduction of SNR and CNR while diagnostic interpretability is preserved. By reconstruction of low-keV images for example at 50 keV, a significant increase in SNR and CNR can be achieved, which effectively diminishes the effects of the CMD reduction. For EID-CT severe contrast media reduction cannot be recommended, diagnostic confidence was poor in the majority of cases.

## Statements and declarations

YCL is supported by a research grant from Siemens Healthcare GmbH. The other authors have no competing interests to declare that are relevant to the content of this article. No funding was received for conducting this study. The local institutional review board approved this study; a waiver for informed patient consent was approved. All scans were acquired during clinical routine, no scans were performed solely for research purposes. The study was conducted in accordance with the Declaration of Helsinki and its amendments.

## Ethics statement

This study was reviewed and approved by theEthics Committee of the Medical Faculty of the University of Bonn with the approval number 027/23. The Ethics Committee of the Medical Faculty of the University of Bonn waived the need of informed consent.

## Data availability statement

The data associated with the study will be made available on reasonable request.

## CRediT authorship contribution statement

**Yannik C. Layer:** Writing – original draft, Visualization, Validation, Methodology, Investigation, Formal analysis, Data curation. **Alexander Isaak:** Writing – review & editing, Investigation, Formal analysis. **Narine Mesropyan:** Writing – review & editing. **Patrick A. Kupczyk:** Writing – review & editing. **Julian A. Luetkens:** Writing – review & editing. **Tatjana Dell:** Writing – review & editing. **Ulrike I. Attenberger:** Writing – review & editing, Resources, Funding acquisition. **Daniel Kuetting:** Writing – review & editing, Supervision, Project administration, Methodology, Conceptualization.

## Declaration of competing interest

The authors declare the following financial interests/personal relationships which may be considered as potential competing interests: Yannik Christian Layer reports a relationship with 10.13039/501100004830Siemens Healthcare AG that includes: funding grants.

## Abbreviations

BMI: Body mass index
CI: Confidence interval
CMD: Contrast media dose
CNR: Contrast-to-noise ratio
CT: Computed tomography
DLP: Dose length product
EID-CT: Energy-integrating detector CT
HU: Hounsfield units
ICC: Intraclass correlation coefficient
IQR: Interquartile range
PCD-CT: Photon-counting detector CT
ROI: Region of interest
SNR: Signal-to-noise ratio
SD: Standard deviation
VMI: Virtual monoenergetic images

## References

[1] Christiansen C. (2005). X-ray contrast media--an overview. Toxicology.

[2] Damilakis J. (2021). CT dosimetry: what has been achieved and what remains to Be done. Invest. Radiol..

[3] Huynh K., Baghdanian A.H., Baghdanian A.A. (2020). Updated guidelines for intravenous contrast use for CT and MRI. Emerg. Radiol..

[4] Sengar A., Vijayanandan A. (2021). Comprehensive review on iodinated X-ray contrast media: complete fate, occurrence, and formation of disinfection byproducts. Sci. Total Environ..

[5] Dekker H.M., Stroomberg G.J., Prokop M. (2022). Tackling the increasing contamination of the water supply by iodinated contrast media. Insights Imaging.

[6] Jensen L. (2022).

[7] ACR Committee on Drugs and Contrast Media (2022).

[8] Lell M.M., Jost G., Korporaal J.G. (2015). Optimizing contrast media injection protocols in state-of-the art computed tomographic angiography. Invest. Radiol..

[9] Willemink M.J., Persson M., Pourmorteza A. (2018). Photon-counting CT: technical principles and clinical prospects. Radiology.

[10] Thor D., Brismar T.B., Fischer M.A. (2015). Low tube voltage dual source computed tomography to reduce contrast media doses in adult abdomen examinations: a phantom study. Med. Phys..

[11] Rajendran K., Petersilka M., Henning A. (2022). First clinical photon-counting detector CT system: technical evaluation. Radiology.

[12] Sawall S., Klein L., Amato C. (2020). Iodine contrast-to-noise ratio improvement at unit dose and contrast media volume reduction in whole-body photon-counting CT. Eur. J. Radiol..

[13] Flohr T., Petersilka M., Henning A. (2020). Photon-counting CT review. Phys. Med..

[14] Booij R., van der Werf N.R., Dijkshoorn M.L. (2022). Assessment of iodine contrast-to-noise ratio in virtual monoenergetic images reconstructed from dual-source energy-integrating CT and photon-counting CT data. Diagnostics.

[15] Wrazidlo R., Walder L., Estler A. (2022). Radiation dose reduction in contrast-enhanced abdominal CT: comparison of photon-counting detector CT with 2nd generation dual-source dual-energy CT in an oncologic cohort. Acad. Radiol..

[16] Hagen F., Hofmann J., Wrazidlo R. (2022). Image quality and dose exposure of contrast-enhanced abdominal CT on a 1st generation clinical dual-source photon-counting detector CT in obese patients vs. a 2nd generation dual-source dual energy integrating detector CT. Eur. J. Radiol..

[17] Emrich T., O'Doherty J., Schoepf U.J. (2023). Reduced iodinated contrast media administration in coronary CT angiography on a clinical photon-counting detector CT system: a phantom study using a dynamic circulation model. Invest. Radiol..

[18] Higashigaito K., Mergen V., Eberhard M. (2023). CT angiography of the aorta using photon-counting detector CT with reduced contrast media volume. Radiology: Cardiothoracic Imaging.

[19] Cavallo J.J., Pahade J.K. (2022). Practice management strategies for imaging facilities facing an acute iodinated contrast media shortage. AJR Am. J. Roentgenol..

[20] van der Molen A.J., Reimer P., Dekkers I.A. (2018). Post-contrast acute kidney injury - Part 1: definition, clinical features, incidence, role of contrast medium and risk factors : recommendations for updated ESUR Contrast Medium Safety Committee guidelines. Eur. Radiol..

[21] Baert A.L., Knauth M., Thomsen H.S. (2009). Contrast Media: Safety Issues and ESUR Guidelines.

[22] Rudnick M.R., Leonberg-Yoo A.K., Litt H.I. (2020). The controversy of contrast-induced nephropathy with intravenous contrast: what is the risk?. Am. J. Kidney Dis..

[23] Perrin E., Jackson M., Grant R. (2018). Weight-adapted iodinated contrast media administration in abdomino-pelvic CT: can image quality be maintained?. Radiography.

[24] Nowak A., Pacek G., Mrozik A. (2020). Transformation and ecotoxicological effects of iodinated X-ray contrast media. Rev. Environ. Sci. Biotechnol..

[25] Xu Z., Li X., Hu X. (2017). Distribution and relevance of iodinated X-ray contrast media and iodinated trihalomethanes in an aquatic environment. Chemosphere.

[26] Zhang W., Fourcade F., Amrane A. (2023). Removal of iodine-containing X-ray contrast media from environment: the challenge of a total mineralization. Molecules.

[27] Masuda T., Nakaura T., Funama Y. (2017). Aortic and hepatic contrast enhancement during hepatic-arterial and portal venous phase computed tomography scanning: multivariate linear regression analysis using age, sex, total body weight, height, and cardiac output. J. Comput. Assist. Tomogr..

